# Readiness Visual Analog Scale: A Simple Way to Predict Post-Stroke Smoking Behavior

**DOI:** 10.3390/ijerph120809536

**Published:** 2015-08-13

**Authors:** Przemyslaw Bienkowski, Pawel Zatorski, Agata Glebicka, Anna Scinska, Iwona Kurkowska-Jastrzebska, Magdalena Restel, Jerzy Samochowiec, Danuta Ryglewicz, Halina Sienkiewicz-Jarosz

**Affiliations:** 1 Department of Pharmacology, Institute of Psychiatry and Neurology, 02-957 Warsaw, Poland; E-Mails: bienkow@ipin.edu.pl (P.B.); pzatorski@wp.pl (P.Z.); aglebicka@ipin.edu.pl (A.G.); 2 1st Department of Neurology, Institute of Psychiatry and Neurology, 9 Sobieskiego St., 02-957 Warsaw, Poland; E-Mails: scinska@yahoo.com (A.S.); magda.restel@gmail.com (M.R.); ryglew@ipin.edu.pl (D.R.); 3 2nd Department of Neurology, Institute of Psychiatry and Neurology, 02-957 Warsaw, Poland; E-Mail: ikurkowska@ipin.edu.pl; 4 Department of Psychiatry, Pomeranian Medical University, 70-204 Szczecin, Poland; E-Mail: samoj@pum.edu.pl

**Keywords:** cigarette smoking, ischemic stroke, secondary prevention, readiness to quit, visual analog scale

## Abstract

Background and Purpose: The aim of the present study was to assess a relationship between readiness to quit and post-stroke smoking behavior. Methods: Eighty-six active smokers with first-ever ischemic stroke were recruited in a tertiary-care stroke unit. The question “Are you ready to quit smoking within the next month?” with yes/no responses and the 10-cm readiness visual analog scale (VAS) was administered during the anti-smoking intervention. Smoking status was verified at the 3- and 12-month follow-up. Results: The readiness VAS score at hospitalization was significantly lower in patients classified as smokers as compared to patients classified as non-smokers. The readiness score <5 cm was a significant predictor of smoking at the 3-month (OR, 7.3) and 12-month follow-up (OR, 4.9). Conclusions: The present results suggest that the readiness VAS can be used as a simple and inexpensive instrument for early identification of patients who continue to smoke after stroke.

## 1. Introduction

The transtheoretical model (TTM) of behavior change was developed to assess the readiness to change smoking behavior and a chance for smoking cessation [1,2]. Readiness to quit predicts smoking abstinence in smokers recruited in clinical and non-clinical settings and the assessment of readiness is an important part of anti-smoking interventions [1,2,3]. Although smoking cessation is considered an effective method of secondary stroke prevention [4,5], the concept of readiness to quit has not been tested in patients who smoked before stroke onset, as yet.

Our preliminary study [5] has shown that up to 100% of hospitalized patients who had smoked before stroke onset answered “yes” to a question “Are you ready to quit within the next month?” Thus, it is possible that quantitative differences in readiness to quit are masked when simple questions based on the TTM are administered to stroke survivors [1,2]. When considering the use of complex multi-item instruments to measure readiness to change [1,2], one should bear in mind that the lack of time and training are important barriers to providing anti-smoking interventions by health care professionals [3]. Hence, in the present pilot study, we decided to use a vertical visual analog scale [6] to quantify readiness to quit in smokers with first-ever ischemic stroke.

## 2. Design

The study was carried out in accordance with the Declaration of Helsinki and its protocol was approved by a local ethics committee. Written informed consent was obtained from all participants. 

The protocol of the study was similar to that used in our previous studies on stroke patients who had smoked before stroke onset [4,5]. Active smokers with first-ever ischemic stroke were selected from patients admitted between 2011 and 2012 to a tertiary-care stroke unit located in an urban area. Active smoker was defined as having smoked within the week before hospitalization. One hundred and ten patients were invited to participate. Twenty-four potential participants were excluded because they declined to participate (n = 10 patients), had hemorrhagic stroke, a previously-diagnosed brain lesion, severe concomitant neurological diseases, psychosis, consciousness disturbances, other than nicotine drug use disorders, and/or severe aphasia making them unable to cooperate. The final sample included 86 Caucasians (see Table 1) who were relatively young and independent as compared to European first-ever stroke patients [7].

**Table 1:** Baseline characteristics of 86 smokers with first-ever ischemic stroke ^*^.

Age (year)	58.0 ± 9.7 ^†^
Women (%)	28.0
Body Mass Index (kg/m²)	27.0 ± 4.5
University degree (%)	15.1
Employed before stroke onset (%)	52.3
Living with their families (%)	86.0
Number of concomitant medical states	2.8 ± 1.7
Barthel Index	17.3 ± 4.9
NIHSS	3.1 ± 2.8
Age of smoking initiation (year)	20.0 ± 3.8
Number of cigarettes per day	23.9 ± 10.3
FTND	5.1 ± 1.9
Readiness VAS (0–10 cm)	6.8 ± 3.4

NIHSS, the National Institutes of Health Stroke Scale; FTND, Fagerström Test for Nicotine Dependence; ^*^ assessed at hospitalization; **^†^** means ± SD.

The baseline assessment was completed during hospitalization, between the 10th and 14th day after stroke. Severity of nicotine dependence was assessed by the Fagerström Test for Nicotine Dependence (FTND) [3,4]. Each participant received a brief smoking cessation intervention in accordance with the “5 A’s” model [3]. The question “Are you ready to quit smoking within the next month?” with yes/no responses and the readiness VAS was administered during the intervention. The readiness VAS was the paper-and-pencil instrument consisting of the 10-cm vertical line, marked at the ends “not at all” and “very much”, and the question “How ready are you to quit smoking right now?” [1,2]. Test-retest reliability of the readiness VAS was evaluated in a separate group of hospitalized smokers with cerebrovascular disorders (n = 20, 30% women). The reliability of readiness scores was good with an intraclass correlation coefficient of 0.85 (*p* < 0.001).

A subject was classified as a non-smoker at a three-month follow-up visit (T1) if he/she reported not smoking for the last four weeks and had an expired-air carbon monoxide level ≤6 parts per million (measured between 1:00 p.m. and 3:00 p.m.) [4,5]. Ten patients were unable to complete the three-month follow-up assessment. After a telephone interview, all of these patients were classified as smokers. Smoking status was verified again through a telephone interview performed by a physician 12 months after stroke (T2). A subject was classified as a non-smoker at T2 if he/she reported not smoking for the last four weeks.

Readiness scores in smokers and non-smokers were compared by the Mann-Whitney U-test. Odds ratios (OR) and 95% confidence intervals (CI) were calculated to assess the association between readiness scores and smoking abstinence. *p* values less than 0.05 were considered significant. Sensitivities and specificities to predict smoking at T1 were calculated for different cut-offs of the readiness VAS score.

## 3. Results

Although all of the patients answered “yes” when asked the question “Are you ready to quit smoking within the next month?”, 57 patients (66.3%) were classified as smokers at T1 and T2. Four patients classified as non-smokers at T1 relapsed between T1 and T2. Four patients achieved abstinence between the two follow-ups. 

The mean (± SD) readiness VAS score at hospitalization in the whole study group was 6.8 ± 3.4 cm (median and interquartile range: 8.5 cm (4.6 cm–9.7 cm)). The readiness VAS score at hospitalization was significantly lower in the patients classified as smokers as compared to the patients classified as non-smokers at T1 (5.9 ± 3.5 cm *vs.* 8.7 ± 2.2 cm) and T2 (6.1 ± 3.6 *vs.* 8.4 ± 2.3; *p* < 0.01). The readiness score ≤5 cm was a significant predictor for continued smoking at T1 (OR, 7.3; 95% CI, 2.0 to 26.8) and T2 (OR, 4.9; 95% CI, 1.5 to 15.9; *p* < 0.01). 

Low readiness scores at hospitalization showed relatively high specificities, but moderate sensitivities, for identifying smokers at T1. For cut-off point ≤3 cm sensitivity, specificity, and positive predictive value (PPV) was 22.8%, 93.1%, and 86.7%, respectively. For cut-off point ≤5 cm sensitivity, specificity, and PPV was 45.6%, 89.6%, and 89.6% respectively. For cut-off point ≤9 cm sensitivity, specificity, and PPV was 75.4%, 69.0%, and 82.7%, respectively. 

## 4. Discussion

Although the VASs are used for evaluation of mood states and pain intensities in various populations, including stroke patients [6], this is the first study on the utility of the readiness VAS for predicting smoking abstinence after stroke. Our major finding is that low readiness scores identify stroke patients being at risk of continued smoking at the 3- and 12-month follow-up. The present study tend to indicate that qualitative measures of readiness to quit, like yes/no questions [3,8], can mask quantitative differences between stroke patients and make identification of subjects who are at risk of continued smoking difficult.

The mean readiness VAS score was relatively high and all of the patients declared readiness to quit within the next month when asked the yes/no question. In this respect, our findings fit well to data reported by Suñer-Soler *et al.* [8]. The latter authors have estimated that more than 85% of smokers with ischemic stroke did not consider smoking as a problem shortly before stroke onset. This proportion decreased to 19% during hospitalization [8]. Thus, it seems that stroke, like other life-threatening diseases [9], leads to an immediate increase in readiness to quit.

The present study involves some limitations, including a relatively small sample size and the fact that all the patients were recruited in one hospital. The 12-month follow-up was based on the telephone interview without CO measurement. Patients with hemorrhagic stroke and more severe neurological deficits were excluded.

## 5. Conclusions

Given the debilitating impact of stroke on individuals and societies [7,10], further studies on barriers to quit in stroke survivors are urgently needed, with a special emphasis on identification of patients who are at risk of continued smoking. Our results suggest that the readiness VAS may help to identify these patients in a simple and inexpensive way. 
